# Characterization and phylogenetic analysis of the complete mitochondrial genome of the polychaete, *Melinna cristata*

**DOI:** 10.1080/23802359.2021.1962755

**Published:** 2021-09-24

**Authors:** Sang-Eun Nam, Somyeong Lee, Jae-Sung Rhee

**Affiliations:** a Department of Marine Science, College of Natural Sciences, Incheon National University, Incheon, South Korea; b Research Institute of Basic Sciences, Incheon National University, Incheon, South Korea; c Institute of Green Environmental Research Center, Incheon, South Korea

**Keywords:** Complete mitogenome, Terebellida, Melinnidae, *Melinna cristata*, polychaete

## Abstract

In this study, we report the complete mitogenome sequence of the polychaete, *Melinna cristata* (Sars, 1851). The circular *M. cristata* mitochondrial genome is 15,696 bp in length and has an AT content of 66%. As in other polychaetes, the genome has 13 protein-coding genes (PCGs), two ribosomal RNA (rRNA) genes, 22 transfer RNA (tRNA) genes, and a non-coding region. Gene composition and their order in the *M. cristata* mitochondrion are identical to the Terebelliformia mitogenomes. A maximum-likelihood gene tree based on the *M. cristata* mitogenome combined with previously published Sedentaria and Errantia mitogenomes revealed that *M. cristata* forms a clade with two Terebelliformia species.

The segmented worms, Annelida comprise one of the most complex taxa with remarkable ecological diversity, broad life strategies, and highly derived morphologies (Struck et al. 2011; Andrade et al. 2015). The annelid species are dominant members in a significant part of the endo- and epibenthos of the marine environment, although some species can be found as a holopelagic species. The diversity of Annelida is classified by phylogenomic analyses into two large monophyletic groups, Sedentaria and Errantia (Struck et al. 2011; Kvist and Siddall 2013; Weigert et al. 2014), although the phylogenetic relationship on Annelida remained controversial for a long time. The marine polychaete, *Melinna cristata* (Sars, 1851) inhabits soft sediments from intertidal to deep sea with depths between 40 and 550 m and exhibits a wide geographical distribution, ranging from the entire Norwegian coast to the Svalbard and the Barents Sea (Mackie and Pleijel 1995). The subfamilies Melinninae and Ampharetinae have traditionally been placed in the family Ampharetidae, suborder Terebellomorpha, and order Terebellida. However, a recent study, based on 12,674 orthologous gene information and morphological data, strongly suggested that the former subfamily Melinninae is the sister to another Terebellomorpha family Terebellidae and does not group together with Ampharetinae (Stiller et al. 2020). Subsequently, the subfamily Melinninae is now suggested to family level Melinnidae and the subfamily Ampharetinae becomes Ampharetidae (Stiller et al. 2020). Since mitogenome information on Terebelliformia is limited, as only two whole mitogenomes, *Pista cristata* (Terebellida; Terebellidae) and *Terebellides stroemii* (Terebellida; Trichobranchidae) have been registered at NCBI GenBank, the complete mitogenome sequence of *M. cristata* will serve as an essential resource for understanding the phylogenetic relationship and evolutionary history of Terebelliformia.

A specimen of *M. cristata* was collected from the Beaufort Sea (69°52′N, 139°03′W) in 2017 using a remotely operated underwater vehicle (ROV) belonging to the Monterey Bay Aquarium Research Institute (MBARI). The sample was deposited in the Korea Polar Research Institute (Species ID: Annelid-03; Specimen ID: KOPRI-Benthos-24). Genomic DNA was isolated from the muscle tissue of *M. cristata* using a DNeasy Blood and Tissue kit (Qiagen, Hilden, Germany) according to the manufacturer’s instructions. Next-generation sequencing was conducted to obtain a circular mitogenome using the protocols based on a previous study (Park et al. 2020). TruSeq DNA Sample Preparation Kit (Illumina, San Diego, CA) was used for sequencing using the Illumina HiSeq sequencer. The sequencing library was prepared by random fragmentation of the DNA sample, followed by 5′ and 3′ adapter ligation. Raw reads were obtained from the sample that passed the quality control check in the Illumina HiSeq platform (Illumina, San Diego, CA) at Macrogen, Inc. (Seoul, South Korea). Adapter sequences, low quality reads, reads with >10% of unknown bases, and ambiguous bases were removed to obtain high quality assembly. After the quality check process, a total of 19,392,958 filtered reads were obtained from 34,246,812 raw reads. Thereafter, *de novo* assembly was conducted with various k-mers using SPAdes (Bankevich et al. 2012), and a circular contig of the *M. cristata* mitogenome was obtained. The resulting contig consensus sequence was annotated using MITOS2 (Bernt et al. 2013) and tRNAscan-SE 2.0 (Lowe and Eddy 1997). Further, BLAST searches confirmed the identity of the genes (http://blast.ncbi.nlm.nih.gov).

The assembled circular mitogenome of *M. cristata* was 15,696 bp in length (GenBank accession no. MW542504), containing 13 protein-coding genes (PCGs), 22 transfer RNAs (tRNAs), two ribosomal RNAs (rRNAs), and one non-coding region. The nucleotide composition was significantly biased toward A + T nucleotides (66%), as the percentages of A, T, C, and G were 36.4%, 29.9%, 12.9%, and 20.8%, respectively. The overall genomic architecture of the *M. cristata* mitochondrion is identical to other mitogenomes of the Terebelliformia (e.g. *Pista cristata*, *Terebellides stroemii*).

A maximum-likelihood (ML) phylogenetic hypothesis was established using sequence data from the concatenated set of the whole 13 PCGs of the *M. cristata* mitogenome, five published mitogenomes belonging to Sedentaria, 17 mitogenomes involved in Errantia, and two Sipuncula species as an outgroup (Figure 1). JModelTest ver. 2.1.10 (Darriba et al. 2012) was used to select the best substitution model and a substitution model (HKY + G+I) was applied to perform a ML method in the PhyML 2.4.5 (Guindon and Gascuel 2003) with 1000 bootstrap replicates. Overall, the relationship between major families in Annelida followed a well-established phylogeny (Weigert and Bleidorn 2016), and families belonging to Terebelliformia also exhibit reliable phylogenetic relationships. The *M. cristata* clustered together with *Terebellides stroemii* (Terebellida, Trichobranchidae) with a bootstrap support of 99% and formed a sister group with *Pista cristata* (Terebellida, Terebellidae). Molecular phylogenetic relationship in families belonging to Sedentaria is still controversial due to limited information of whole mitogenomes, although Sedentaria is a species-rich group as a member of Pleistoannelida together with its sister taxa Errantia. Based on the recent robust phylogenomic result constructed with transcriptome and morphological data, Melinnidae was placed as the sister group to Terebellidae and further formed with Trichobranchidae (Stiller et al. 2020), which differs from our result in which Melinnidae firstly clusters with Trichobranchidae, possibly due to the absence of incorporation of additional phylogenomic materials in this study. Nevertheless, this new mitochondrial genome will be useful for smaller scale phylogenies within annelida.

**Figure 1.**
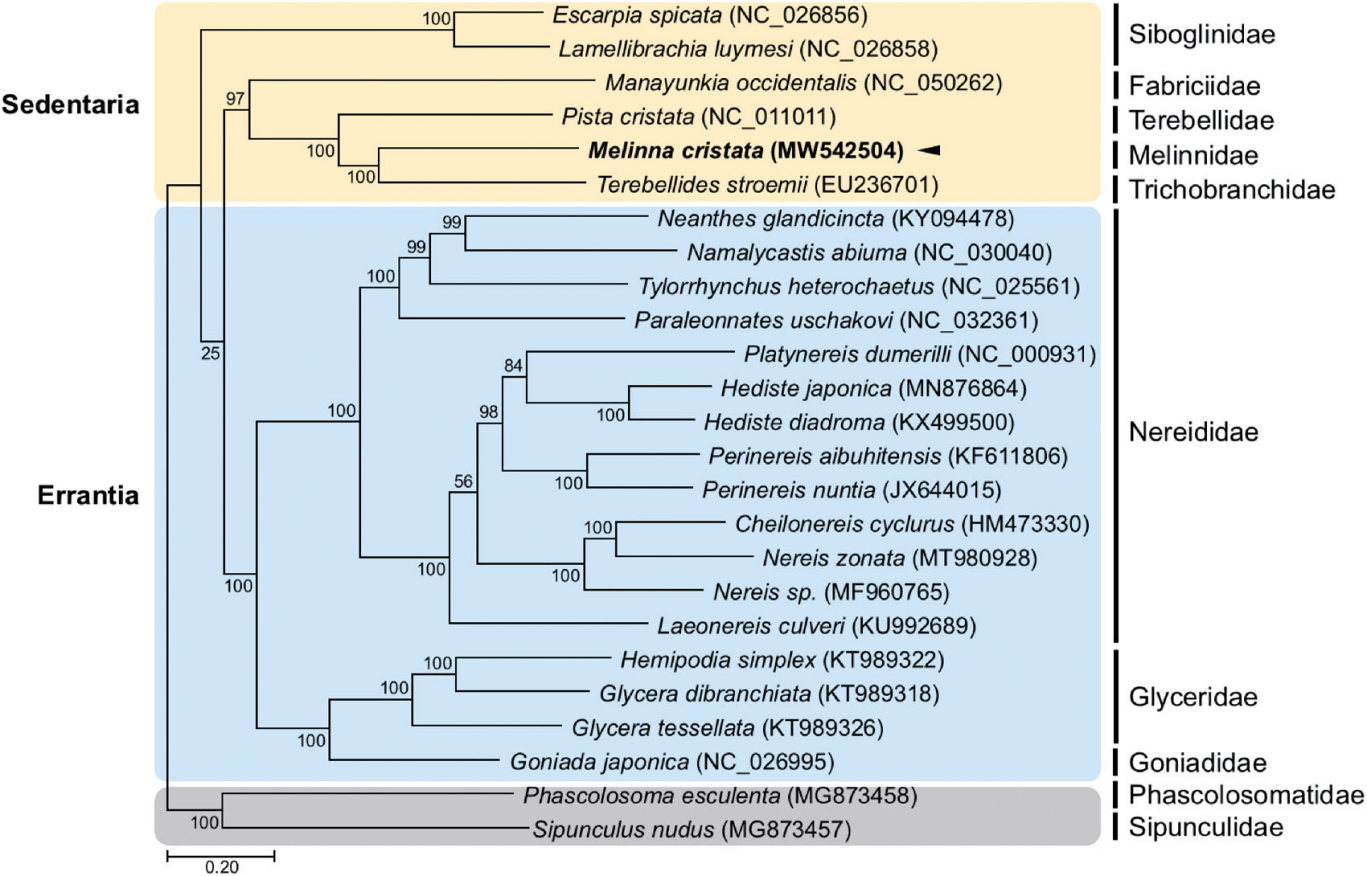
Maximum-likelihood (ML) phylogeny of 6 published mitogenomes from Sedentaria including *M. cristata* and 17 registered mitogenomes of Errantia species, and two Sipuncula species as an outgroup based on the concatenated nucleotide sequences of protein-coding genes (PCGs). Numbers on the branches indicate ML bootstrap percentages. DDBJ/EMBL/GenBank accession numbers for published sequences are incorporated. The black triangle means the polychaete analyzed in this study.

## Data Availability

BioProject, SRA, and BioSample accession numbers are https://www.ncbi.nlm.nih.gov/bioproject/PRJNA695143 and https://www.ncbi.nlm.nih.gov/biosample/SAMN17602389, respectively. The data that support the findings of this study are openly available in the National Center for Biotechnology Information (NCBI) at https://www.ncbi.nlm.nih.gov, with an accession number MW542504.
